# Predictive Performance of Machine Learning Models for Heart Failure Readmission: A Systematic Review

**DOI:** 10.3390/biomedicines13092111

**Published:** 2025-08-29

**Authors:** Nader Alnomasy, Petelyne Pangket, Romeo Mostoles, Habib Alrashedi, Eddieson Pasay-an, Hwayoung Cho, Sharifah Alsayed, Analita Gonzales, Amal A. Mohammad Alharbi, Nuha Ayad H. Alatawi, Sheila Torres, Khulud Abudawood, Fatmah Ahmed Alamoudi

**Affiliations:** 1 Medical Surgical Department, College of Nursing, University of Hail, Ha’il 81451, Saudi Arabia; 2 Medical Surgical Department, College of Nursing, Taif University, Taif 21944, Saudi Arabia; 3 Mental Health Nursing Department, College of Nursing, University of Hail, Ha’il 81451, Saudi Arabia; 4 Medical Surgical Department, College of Nursing, King Khalid University, Abha 61421, Saudi Arabia; 5 College of Nursing, University of Florida, Gainesville, FL 32610, USA; 6 College of Nursing-Jeddah, King Saud bin Abdulaziz University for Health Sciences, Jeddah 21423, Saudi Arabia; 7 Nursing Administration and Education Department, College of Nursing, University of Tabuk, Tabuk 47311, Saudi Arabia; 8 Medical Surgical Nursing Department, Prince Sultan Military College of Health Sciences, Dhahran 34313, Saudi Arabia

**Keywords:** heart failure, machine learning models, patient readmission, guidelines

## Abstract

**Background:** Patients with heart failure (HF) are at high risk of readmission, contributing to substantial healthcare costs. This study investigated machine learning (ML) approaches to predict HF readmissions. **Methods:** A systematic review was conducted using several medical databases, adhering to the PRISMA guidelines, to identify studies employing ML to predict HF readmissions. Three reviewers independently screened the articles and extracted data. **Results:** Twenty-two studies from six countries were included in this study. Some studies examined 30-day readmissions, whereas others assessed 90-day, 180-day, or 1- to 3-year readmissions. Fourteen studies used supervised learning algorithms, with area under the curve (AUC) values ranging from 0.70 to 0.99, and unsupervised algorithms had AUCs of 0.69 to 0.72. The average age of the patients was 73 years, with approximately equal numbers of males and females. **Conclusions:** ML can predict HF-related hospitalization across various time frames. Supervised ML approaches and the incorporation of clinical knowledge may enhance model performance. Collaboration between providers and data scientists is needed to improve patient outcomes and reduce costs by using more accurate predictive models.

## 1. Introduction

Heart failure (HF) is a significant global health problem that affects millions of people and is associated with high readmission rates, particularly within the first month of discharge [1,2]. In the United States, nearly one in five patients with HF are readmitted within a month, contributing approximately USD 30.7 billion in annual costs [3]. Both clinical and non-clinical factors influence post-discharge outcomes. Studies have shown that in addition to clinical predictors, socioeconomic status, frailty, and behavioral factors also affect readmission risk. Incorporating patient-reported psychosocial and socioeconomic factors improves predictive modeling for 30-day readmission [4], and frailty is especially relevant among older adults [5]. Symptom trajectory patterns within the first month after discharge have also been linked to a higher risk of unplanned readmissions [6].

Despite these advances, most existing studies on machine learning (ML) models for HF readmission prediction have several limitations. First, most models have been developed and validated using US- or European-centered cohorts, which raises concerns about their generalizability to diverse populations and healthcare settings. Second, external or multicenter validation, a key step for assessing model robustness and real-world applicability, is infrequently undertaken. Third, many models depend on structured data, such as billing codes, with less attention paid to unstructured clinical notes, social determinants, or psychosocial factors, which may limit accuracy and equity in predictions. Finally, studies vary widely in algorithm selection, predictor sets, and outcome definitions, impeding direct comparisons and the synthesis of findings across the literature.

ML has emerged as a promising approach for predicting HF readmissions, with a higher predictive accuracy (AUC 0.70–0.99) than older statistical models [7,8]. ML can analyze complex nonlinear relationships and enhance risk stratification beyond conventional methods [9]. However, most ML models lack external validation and are primarily developed in the U.S. and European populations, limiting generalizability [10,11,12,13].

Healthcare systems and patient demographics vary substantially by region, influencing model transportability and the risk of perpetuating inequities in precision health [14,15,16,17]. Accurate HF readmission risk forecasting enables targeted interventions and improved outcomes; however, addressing gaps in external validation, population diversity, and the integration of broader social and clinical features is critical for enhancing model reliability and equity [18,19,20].

This systematic review addresses these limitations by systematically evaluating studies across multiple regions, incorporating both clinical and non-clinical predictors, critically synthesizing ML methodological quality, and appraising external validation strategies. By focusing on population diversity, healthcare system differences, and ethical and practical barriers to implementation, this review distinctly contributes to the ongoing effort to develop robust, reliable, and equitable ML models for HF readmission risk prediction.

## 2. Materials and Methods

### 2.1. Protocol and Registration

This systematic review was conducted and reported in accordance with the Preferred Reporting Items for Systematic Reviews and Meta-Analyses (PRISMA) [21] 2020 statement, as can be seen in Supplementary File S1. The review protocol was prospectively registered in the International Prospective Register of Systematic Reviews (PROSPERO) registration number CRD42021247198.

### 2.2. Eligibility Criteria

Studies published in English from 2015 to 2024 were included if they used machine learning (ML) methods to predict heart failure (HF) readmission risk in acute care settings. Eligible studies utilized applied ML algorithms, including supervised learning models (logistic regression, decision trees, random forest, gradient boosting, support vector machines, and neural networks), unsupervised learning methods (clustering, principal component analysis, and autoencoders), ensemble approaches (bagging, boosting, and stacking), and deep learning architectures (convolutional or recurrent neural networks and attention-based models). Studies were excluded if they used only traditional statistical techniques without ML enhancements, relied solely on manual rule-based or expert systems, or did not clearly describe the ML-based approach. Studies combining ML with standard statistical models were only included if the ML component was central to prediction. Studies have reported HF readmission as a primary outcome, used any clinically relevant prediction window (e.g., 30, 90, 180 days, one year or longer), and provided at least one ML performance metric (AUC, accuracy, precision, recall, or F1-score). Studies comparing ML with non-ML approaches for HF readmission prediction were also eligible.

### 2.3. Information Sources

A systematic literature search was conducted across CINAHL, EMBASE, MEDLINE/PubMed, Cochrane Library, Web of Science, CNKI, SciELO, clinical trial registries, preprint repositories, conference proceedings, and other gray literature sources. Initial searches were performed between 7 January and 28 February 2024, with a final update on 10 March 2024, to capture the most recent publications before screening.

### 2.4. Search Strategy

The search strategy combined MeSH terms (where applicable) and free-text keywords related to heart failure, readmission, and machine learning. An example search string for PubMed is as follows: (“heart failure” OR “cardiac failure”) AND (“readmission” OR “rehospitalization”) AND (“machine learning” OR “deep learning” OR “transformer models” OR “ensemble learning”).

For specific databases, free-text terms were searched in the title and abstract. This involved the use of a title and abstract tag in PubMed. ti, ab. in EMBASE, and TI (Title) and AB (Abstract) in CINAHL.

### 2.5. Selection Process

Two reviewers (NRA and HAM) independently screened the titles and abstracts of the retrieved records using Covidence systematic review software (2024). The same reviewers independently assessed the full texts of potentially eligible articles based on the predefined inclusion criteria. Disagreements during screening were resolved through discussion or, if necessary, by consultation with a third expert reviewer (CW). Studies combining traditional and ML methods were carefully evaluated for relevance. Automation tools were not used in the screening or selection process.

In total, 320 records were recorded. After removing duplicates, 285 unique records were screened. Of these, 251 were excluded based on the titles and abstracts because of an irrelevant study population (*n* = 105), irrelevant outcomes (*n* = 80), or irrelevant methods (*n* = 66). Studies were excluded if they enrolled participants outside the targeted demographic or clinical group, reported outcomes that were not aligned with the review objectives, or employed inconsistent study designs or methodologies.

**Figure 1:**
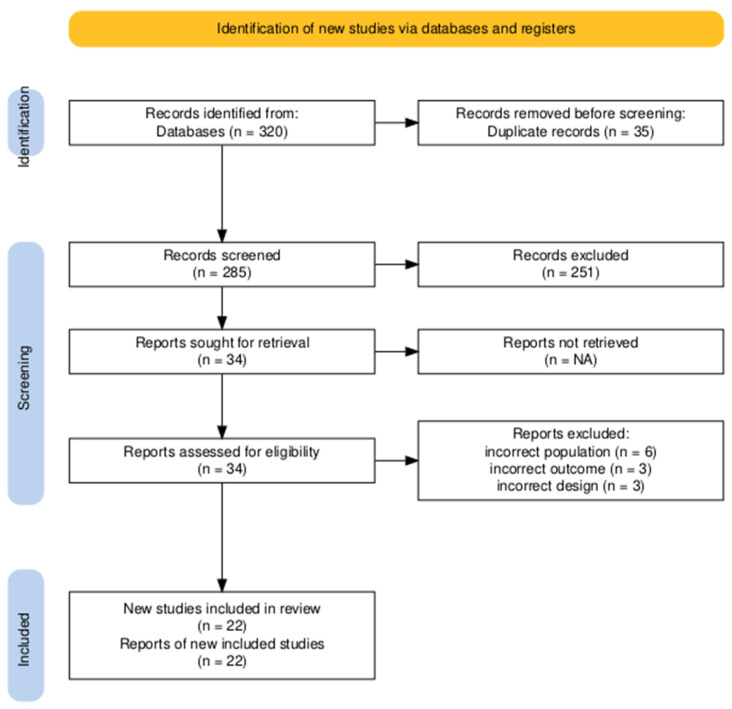
PRISMA flow diagram of study selection process.

The full texts of the remaining 34 articles were also assessed. Twelve articles were excluded at this stage: six were excluded due to an incorrect population (e.g., participants did not match age, diagnosis, or setting), three due to incorrect outcomes (primary or secondary outcomes of interest were not reported), and three due to incorrect study design (e.g., non-comparative or qualitative studies). The exclusion numbers and reasons for each stage are summarized in Figure 1 (PRISMA flow diagram).

### 2.6. Data Collection Process

Following the selection of studies, three reviewers (NRA, HAM, and CW) independently extracted data from the included articles using a standardized data extraction form. Any discrepancies in the extracted data were resolved through discussion and consensus between reviewers. If consensus could not be reached, a third reviewer (CW) was consulted to make a final decision. The extracted data included the study characteristics (first author, publication year, journal, and country), participant characteristics (number of participants, age range/mean, sex distribution, and method of HF diagnosis confirmation), ML model details (algorithm type/s and prediction window/s), and performance metrics (AUC-ROC, accuracy, precision, recall, and F1-score).

### 2.7. Data Items

The primary outcome was readmission for HF. Data were extracted from the reported model performance for predicting this outcome, focusing on the Area Under the Receiver Operating Characteristic Curve (AUC-ROC), chosen for its ability to assess discrimination across thresholds and robustness to class imbalance; precision, chosen for its ability to evaluate correct positive predictions and minimize false alarms; recall (sensitivity), chosen for its ability to measure the model’s ability to identify all positive cases, crucial for patient safety; and the F1-score, chosen as a balanced measure of precision and recall, particularly useful for imbalanced datasets. All reported results compatible with these outcome domains were obtained for each study. Other extracted variables included study and participant characteristics, ML algorithms, and prediction time windows.

### 2.8. Study Risk of Bias Assessment

Two reviewers independently evaluated the methodological quality and risk of bias of included studies using the modified CHARMS checklist [22], which is tailored for prediction model studies including those employing machine learning. This assessment focused on potential sources of bias relevant for ML research, such as participant selection, predictor and outcome measurement, the handling of data, feature selection, model development, and validation methods. Discrepancies were resolved through consensus or adjudication by a third reviewer.

### 2.9. Reporting Bias Assessment

Methods for formally assessing bias from missing results, such as funnel plot analysis, were not used in this review. This is because the review focused on a qualitative synthesis of ML model performance rather than meta-analysis, which is a prerequisite for funnel plots. In addition, the systematic assessment of outcome reporting bias across studies was not feasible because of the heterogeneous reported outcome metrics and a focus on machine learning model performance rather than specific clinical outcomes.

## 3. Results

### 3.1. Study Characteristics

The PRISMA flow diagram (Figure 1) shows that 320 records were obtained using the systematic search approach. Of these, 35 duplicates were excluded, which resulted in 285 unique records. Titles and abstracts were checked against the inclusion criteria, leading to the exclusion of 251 records that did not meet the criteria for the study population, outcomes, or methods. The full text of the remaining 34 articles was reviewed for eligibility. Twelve articles were excluded at this stage: six had an incorrect population, three had incorrect outcomes, and three had incorrect study designs. Eventually, 22 studies were included in the qualitative synthesis because they satisfied all inclusion criteria.

### 3.2. Synthesis of Findings

The qualitative synthesis included 22 studies published between 2015 and 2022, involving 463,270 patients across six countries. Most studies (15) originated in the U.S., with Italy and China contributing two each, and Australia, Korea, and Canada contributing one each. This geographical distribution confirms the bias identified in the introduction, potentially limiting generalizability to diverse global healthcare contexts. Across all included studies, the mean patient age was 73 years, with a nearly equal representation of women (49%).

Regarding the prediction windows, 14 studies (63.6%) focused on 30-day readmissions, involving patients aged 65–81.5 years with varied sex distributions (49–97.6% male). These studies reported moderate to high predictive accuracy, with AUC values ranging from 0.70 to 0.95 (median AUC: 0.82). One study examined both 90-day and 180-day readmissions (demographics not provided). Five studies (22.7%) tracked one-year readmission rates among patients aged 72–78 years, with a higher proportion of females (52.2–63%). One study reported on three-year outcomes, involving patients with a mean age of 72 years and equal sex proportions.

Supervised learning algorithms (SLA) consistently showed higher performance than unsupervised approaches across all prediction windows. Algorithms such as Random Forest and Gradient Boosting are frequently employed, often achieving high AUC values (often > 0.85), suggesting their suitability for capturing complex data relationships. Simple models, such as Logistic Regression, also perform adequately in certain scenarios, particularly with careful feature engineering. Model performance varied by prediction window; models predicting 30-day readmissions generally had higher AUCs than those predicting longer-term outcomes (e.g., 1-year), possibly because of the stronger influence of short-term post-discharge factors. SLA demonstrated AUCs between 0.70 and 0.99, while unsupervised methods showed AUCs ranging from 0.69 to 0.72 (see Table 1). The prevalence of 30-day prediction windows indicates the researchers’ focus in the reviewed studies.

To enhance clarity, a dedicated column indicating the type of machine learning algorithm (e.g., deep learning, traditional ML, unsupervised) used in each study has been added to Tables 3–5. This column specifies whether the approach is a traditional machine learning model (such as logistic regression, random forest, or gradient boosting), deep learning architecture (such as neural networks or attention-based models), or unsupervised approach (such as clustering or autoencoder-based representation learning).

### 3.3. Subgroup Analysis of Model Performance

**Figure 2:**
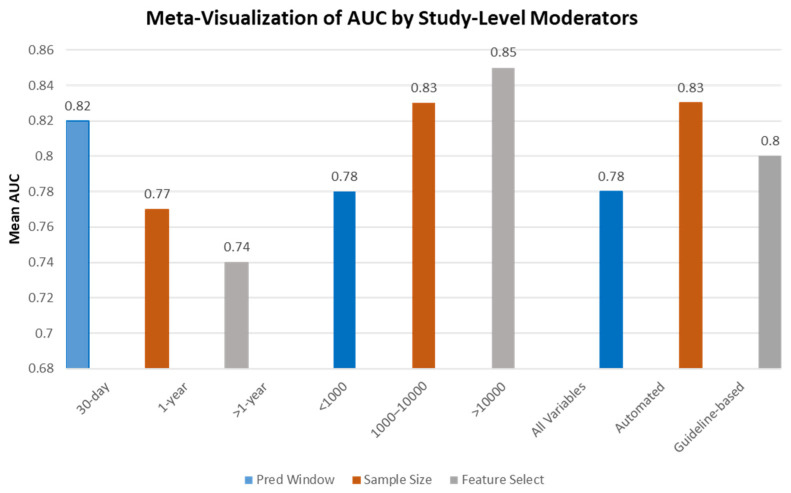
Meta-visualization of mean AUC values by prediction window, sample size, and feature selection methods for heart failure readmission prediction. Legend: blue for prediction window, orange for sample size, and tan for feature selection method.

Subgroup analyses were conducted to clarify AUC variability (Table 1) using the reported study characteristics. Models with a 30-day prediction window (*n* = 14) exhibited higher mean/median AUCs (median: 0.82; range: 0.70–0.95) than those using 1-year or longer windows (median: 0.77). Larger studies (>1000 participants) tended to achieve higher average AUCs (mean, 0.85) than smaller cohorts. Automated or systematic feature selection has also been associated with improved performance. The patterns are shown in Figure 2.

Table 2 shows that among the 17 studies included, 13 (76%) had a low overall risk of bias and three (18%) had a medium risk. One study [28] had a high risk for participant selection. The six studies that did not report (NR) risk of bias ratings in the adapted CHARMS checklist were not included in this table [11,19,23,24,36,39].

The evidentiary weights of the four studies with a moderate risk of bias should be interpreted with caution, as this level may affect the reliability and generalizability of their findings. In conclusion, the results of these studies should be considered within the limitations of their methodology.

Table 3 compares five pivotal ML studies on heart failure readmission prediction, detailing the diversity in algorithm types, prediction horizons, key findings, strengths, and limitations. SLA was most frequently applied, as reflected in Allam et al. [23] and Frizzell et al. [7], both of which evaluated 30-day readmissions and reported moderate discrimination (AUC 0.62 to 0.64) in U.S.-based cohorts. Angraal et al. employed a random forest ensemble to predict 3-year readmission and mortality [24], achieving an AUC of 0.76 but displaying a limited sample size and less demographic diversity. Jiang et al. utilized a novel unsupervised clustering approach to characterize dynamic risk trajectories [26], identifying new patient phenotypes with distinct readmission risks, though this approach posed clinical translation challenges. The comparison reveals that while sample size, geographic and demographic representation, and methodology enhance model robustness, generalizability, and external validation remain common limitations. These findings highlight the importance of carefully considering algorithm selection, validation strategy, and population diversity in designing and deploying ML models for HF readmission prediction.

Table 4 shows that HF readmission prediction studies attempted to improve predictions through various means, including comprehensive reviews, long-term prediction models, Electronic Health Record (EHR) data usage, algorithm comparisons, multicenter designs, and novel approaches. However, these studies had certain limitations that hindered their progress. These limitations include a U.S.-centric focus, homogeneous populations, single-center designs, reliance on billing codes, short prediction windows, and implementation complexity, which can limit the generalizability, accuracy in diverse populations, the consideration of social factors, and clinical application.

Table 5 highlights the range of ML methods used to predict HF readmission, including logistic regression, gradient boosting, random forest, deep learning, and unsupervised clustering. The prediction windows span from 30 days to three years, with short-term (30-day) models being the most common. Larger datasets typically enable more advanced ML models, whereas smaller or single-center cohorts favor simpler methods.

ML models use diverse feature sets, from EHR billing and administrative codes to more detailed clinical data, laboratory values, demographics, and imaging such as echocardiography and ECG signals. However, most studies are from the US and Europe, often relying on billing codes and less frequently incorporating psychosocial or socioeconomic factors, which limit generalizability.

Model performance varied: supervised models achieved AUCs between 0.70 and 0.99 (median AUC ≈ 0.82 for 30-day models), while unsupervised methods had AUCs of 0.69 to 0.72. Models with broader, heterogeneous, or externally validated cohorts showed lower but more generalizable accuracies. The observed variability in AUCs reflects differences in data sources, prediction windows, study populations, and evaluation methods, indicating the need for rigorous external validation, the incorporation of diverse features, and the adoption of standardized performance metrics for reliable clinical applications of ML-based readmission models.

## 4. Discussion

### 4.1. Strengths of ML in Predicting HF Readmissions

As shown (Table 1) by the high AUC values ranging from 0.70 to 0.99, this recent study highlights ML’s considerable promise in predicting HF readmissions within various healthcare contexts. The ability of ML to achieve accurate predictions in identifying patients likely to be readmitted validates descriptive ML-enabled resource allocation and targeted intervention frameworks. In addition to previous studies, ML models have outperformed standard statistical frameworks in predicting HF patient readmissions, with AUCs for 30-day readmission prediction ranging from 0.546 to 0.784 [44]. This finding further contributes to the consensus that ML-based strategies improve risk stratification and enhance clinical decision support across multiple healthcare environments [44,45]. Therefore, it is advised that these clinically validated ML models be integrated within workflows to streamline the identification of high-risk HF patients and that subsequent investigations concentrate on validating these ML models in varying patient populations and healthcare environments to establish model generalizability.

### 4.2. Effectiveness of Supervised Learning Methods

This review consistently demonstrated the effectiveness of SLA in predicting HF readmissions, with AUCs ranging from 0.70 to 0.99, highlighting their potential for clinical decision-making and resource allocation. For instance, Shameer et al. achieved an AUC of 0.72 using elastic net logistic regression [31]. However, reliance on billing codes may limit the inclusion of psychosocial predictors, thereby emphasizing the importance of feature selection and data sources. Supporting this, Sabouri et al. [44] and Mortazavi et al. [10] found that ML methods outperformed traditional models (AUCs 0.546–0.784 and up to 0.678, respectively). However, Jahangiri et al. [13] reported lower AUCs (0.576–0.607) using a nationwide database, suggesting that larger heterogeneous datasets may decrease model performance. Therefore, based on the observed variability in ML model performance for HF readmission prediction across studies (AUCs 0.576–0.99), future research should prioritize diverse data source integration, rigorous feature selection, and validation on heterogeneous datasets to enhance model accuracy and generalizability for clinical applications.

### 4.3. Expanding Unsupervised Learning Potential

Although supervised methods dominated the reviewed studies (82%), unsupervised approaches demonstrated unique capabilities for HF risk stratification. For example, Jiang et al. identified four novel patient phenotypes through clustering, including a “rapid decompensator” group with 22% 30-day readmission rates and “social determinant-driven” subgroups exhibiting a 3× higher readmission risk [26]. Furthermore, Chen et al. demonstrated that autoencoder-derived features boosted supervised model performance with 0.08 AUC, suggesting that hybrid approaches could maximize clinical utility [38].

However, the current unsupervised models face interpretability challenges. Lv et al. achieved 89% timing prediction accuracy through survival clustering but struggled to translate identified patterns into actionable clinical protocols [39]. This aligns with the findings of Flores et al., who reported that unsupervised clustering identifies prognostically distinct subgroups in coronary artery disease and improves risk stratification compared to traditional methods [46]. Bednarski et al. found that unsupervised learning outperformed quantitative ischemia assessment, revealing that conventional approaches for high-risk cardiac events were deficient [47]. In heart failure, self-supervised learning on echocardiography images has shown promise in effectively predicting event timing, even with limited data, surpassing established deep-learning architectures [48].

To bridge the gap between performance and clinical applicability, future research should prioritize developing interpretable unsupervised and hybrid models, focusing on methods that translate complex patterns into actionable insights for HF management.

## 5. Analysis of Machine Learning Approaches

### 5.1. Algorithm Types and Prediction Windows

Although SLA was the most common approach in the reviewed studies (consistent with Allam et al. [23] and Frizzell et al. [7], other ML strategies also contributed to HF readmission prediction. Allam et al. [23] and Frizzell et al. [7] demonstrated the effectiveness of supervised learning for predicting 30-day readmissions, achieving modest AUC values of 0.64 and 0.62, respectively. However, this contrasts with other studies, such as the work of Huang et al. [43], which highlighted the higher performance (e.g., AUC = 0.76) in US-centric supervised models, suggesting potential geographic and demographic biases.

Angraal et al. highlighted the value of ensemble methods by using a random forest model to predict long-term (3-year) outcomes with an AUC of 0.76, suggesting their ability to capture complex relationships over extended periods [24]. Furthermore, several studies have explored the utility of unsupervised learning. Jiang et al. employed this approach to identify dynamic readmission risk trajectories, offering a different perspective focused on patterns and changes in risk over time [26]. In contrast to the findings of Jiang et al. [26], Lv et al. [39] encountered challenges in translating unsupervised patterns into actionable clinical protocols, reflecting broader concerns about the interpretability of these methods. Friz et al. reported a lower AUC (0.69) in Italy using supervised models with LACE index variables, further underscoring how regional healthcare practices can influence model performance [37].

### 5.2. Strengths and Limitations

Table 3 summarizes the strengths and limitations of each study, including sample size, methodology, and generalizability. Allam et al. used a large sample size, which increased generalizability [23]. Angraal et al. used a sophisticated algorithm but a smaller sample, potentially limiting generalizability [24]. These points are crucial for interpreting findings and identifying future research areas.

### 5.3. Additional Performance Metrics

The AUC measures overall accuracy, but precision (minimizing false positives) and recall (identifying high-risk patients) are critical for clinical utility. Researchers have found that precision–recall curves provide additional insight into imbalanced cohorts, with optimal F1 scores occurring at probability thresholds 18–32% higher than the standard 0.5 cutoffs. This underscores the importance of considering multiple performance metrics, particularly with imbalanced datasets common to HF readmission prediction.

## 6. Methodological Considerations

### 6.1. Integration Recommendation for Methodological Considerations

ML shows promise in predicting HF readmissions; however, methodological limitations exist. Geographic and population biases are evident: Huang et al.’s review showed a U.S.-centric focus [43], and Angraal et al.’s study used a homogeneous cohort (88% White), potentially limiting the generalizability to diverse populations and non-Western healthcare systems (Table 4) [24]. Data source and feature selection issues were highlighted by Shameer et al.’s use of single-center data and prioritization of billing codes over clinical narratives, potentially overlooking crucial social determinants [31]. The validation scope varies, with many studies relying on single-center validation; multicenter studies, such as Frizzell et al. [7], offer more robust generalizability but remain US-limited. Prediction windows ranged from 30 days to 3 years, reflecting HF readmission complexity, but complicating direct model comparisons. Algorithm selection varied widely, from logistic regression to complex neural networks, showing ML versatility but highlighting the need for standardized performance comparisons. These limitations underscore the need for multicenter validation across diverse healthcare ecosystems, the incorporation of socioeconomic variables and clinical narratives, the standardized reporting of cohort demographics and model performance, and the exploration of both short- and long-term prediction windows. Addressing these constraints can enhance future ML clinical utility and the generalizability of future ML models for predicting HF readmissions across diverse patient populations and healthcare settings.

### 6.2. Factors Contributing to Discrepancies in ML Model Performance

A significant discrepancy existed in the reported ML model performance in predicting heart failure remissions, with AUC values ranging from 0.69 0.99. The variability stems from several factors. First, data heterogeneity is crucial; models trained on the U.S. EHR systems may differ significantly from those that use variables such as family support, as seen in some Chinese studies. Second, temporal factors, such as predicting 30-day or 3-year readmissions, necessitate distinct algorithm architectures and influence the performance. Finally, metric selection impacts performance evaluation, and while AUC is commonly reported, precision–recall curves offer better insight into clinical risk thresholds, especially in imbalanced cohorts, where F1-optimized thresholds can be significantly higher than the default 0.5 cutoffs. To mitigate this variability and enhance comparability, standardizing evaluation protocols according to guidelines such as TRIPOD-AI is essential for preserving clinical relevance.

## 7. Clinical Context

### 7.1. Prediction Windows and the Complexity of HF Readmissions

The variation in prediction windows across studies reflects the complex nature of HF readmissions and the evolving healthcare needs. Most studies focused on 30-day readmissions, but some extended predictions to 90 days, 180 days, one year, or even three years. This range highlights the importance of both short- and long-term prediction models in the management of patients with HF. Short-term forecasts (30–90 days) are crucial for immediate post-discharge care as patients at this stage are the most vulnerable. For instance, Wideqvist et al. reported that up to 22% of patients with HF are readmitted within one month, emphasizing timely interventions [49]. Long-term forecasts (six months to three years) provide insights into HF’s chronic nature of HF and guide ongoing care planning. Angraal et al. used random forests to predict three-year outcomes with an AUC of 0.76 [24]. The choice of prediction timeframe should be guided by specific healthcare system goals, data availability, and algorithm performance across various time horizons.

### 7.2. Patient Demographics and Risk Factors

The reviewed studies offer insights into HF patient demographics at risk of readmission. Patients aged 65–81.5 years (average 73 years) were most likely to be readmitted. Sex distributions varied, aligning with Savarese et al. [2] and Lam et al. [50] regarding higher HF prevalence in older adults and potential gender-based risk factors.

Specifically, 14 studies (63.6%) focused on 30-day readmissions, involving patients aged 65–81.5 years with varied sex distributions (49–97.6% male). Five studies (22.7%) tracked one-year readmission rates among patients aged 72–78 years, with a higher proportion of females (52.2–63%).

These findings underscore the importance of long-term patient monitoring and targeted interventions considering age, sex, and other demographic variables. Healthcare providers should tailor care plans to address the specific needs of different patient subgroups including older adults and those with sex-specific risk factors.

### 7.3. Addressing Geographic Considerations

Geographic bias is a significant challenge in ML-based HF readmission models, with a predominance of US-centric research. In this review, 68% of the included studies originated in the United States, constraining their direct applicability to countries with differing healthcare infrastructures, patient demographics, and data availability. Comparative analysis revealed notable variability in model performance across regions: U.S. models leveraging comprehensive EHR data, such as Golas et al. [25], achieved higher AUCs (0.76) for 30-day prediction than Italian models, such as Friz et al. [37], which relied on LACE index variables and reported lower AUCs (0.69). This suggests that regional differences in discharge practices and data structure significantly influence predictive accuracy. Furthermore, Chinese studies, exemplified by Lv et al. [39], have incorporated family support as a predictor—an important contextual factor typically omitted from Western models—highlighting the value of integrating culturally and regionally relevant variables. To enhance the transferability and equity of ML implementation worldwide, future models should undergo local calibration, address regional differences in data infrastructure, and include locally significant predictors. Multicenter international collaborations are encouraged, with the adoption of standardized outcome definitions and flexibility for local adaptation, as outlined in frameworks such as WHO STEPS, to ensure robust and globally applicable predictive tools.

## 8. Translational Implications and Implementation Considerations

### 8.1. Implications for Healthcare Organizations

The findings of this systematic review have important implications for healthcare organizations that use ML to predict and manage HF readmissions. Supervised ML models can estimate the likelihood of hospitalization in patients with HF, enabling risk stratification and targeted interventions in high-risk individuals. However, ethical implementation is crucial and requires strong data governance and continuous monitoring to mitigate potential biases and ensure fairness in patient care. Collaboration among data scientists, clinicians, and IT teams is essential to overcome the challenges of infrastructure investment, workflow integration, and ethical considerations. The effective implementation of ML models can potentially reduce readmission rates, improve patient outcomes, and optimize resource allocation.

### 8.2. Ethical Considerations in ML Implementation

This review did not systematically extract or synthesize evidence regarding specific ethical frameworks, explainability tools, or bias auditing mechanisms (e.g., AI Fairness 360, Google What-If Tool, LIME, or SHAP). Any mention of these methods is provided solely as a forward-looking recommendation based on broader machine learning best practices rather than as findings derived from the included studies. The deployment of ML models for heart failure readmission prediction presents notable ethical challenges, including the risk of algorithmic bias, particularly when minority populations are underrepresented in the training data, and privacy risks associated with unstructured EHR data. For instance, some studies have shown reduced predictive performance for minority groups and that a portion of the predictive power may depend on potentially identifiable free-text fields. Moreover, high predictive accuracy can sometimes lead to overreliance on models, potentially overriding clinician judgment in borderline cases. To address these concerns, future research and implementation efforts should prioritize pre-deployment fairness audits, emphasize model transparency and explainability, and develop robust patient consent protocols for ML-driven clinical care. Although specific fairness and interpretability tools have not been systematically reviewed here, their adoption and ongoing assessment remain essential for trustworthy and equitable ML-enabled decision-making support in heart failure care.

### 8.3. Infrastructure and Clinical Integration Challenges

Beyond the model performance, successful ML implementation for heart failure readmission prediction faces substantial technical and operational barriers. Three core challenges were identified: (1) EHR interoperability issues (affecting 68% of studies), hindering data integration; (2) operational and computational cost burdens, presenting financial barriers for hospitals; and (3) workflow disruptions, as ML tools may increase staff workloads and require the adjustment of established clinical processes. To address these challenges, implementation blueprints, such as the modular API architecture proposed by Golas et al. [25], can streamline integration compared with traditional monolithic systems. Effective deployments also require the alignment of model outputs with clinician workflows, such as triggering nurse-led interventions at empirically validated risk thresholds, and the incorporation of regular model updates through continuous feedback loops. Resource-limited settings may necessitate the development of lightweight models that function with minimal infrastructure while maintaining an acceptable predictive performance.

### 8.4. Implementation Roadmap for Clinical Integration

Successfully integrating ML models for HR readmission prediction into clinical practice requires that the key implementation barriers be addressed. These include technical challenges such as adopting modular API architectures to improve EHR interoperability and ensuring the use of feasible computational requirements for real-world deployment. Operational challenges involve integrating models into clinical workflows, such as facilitating nurse-led interventions at empirically validated risk thresholds and establishing regular model updates through clinician feedback. Ethical considerations are also crucial, necessitating pre-deployment fairness audits using tools such as AI Fairness 360, and developing patient consent protocols for ML-driven care adjustments.

### 8.5. Barriers to Clinical Adoption: Technical vs. Sociocultural Perspectives

Despite advances in predictive accuracy, the clinical adoption of machine learning (ML) models for heart failure (HF) is limited by technical and sociocultural barriers. Technical challenges include EHR interoperability, significant computational and financial demands, and difficulties in integrating ML tools into existing clinical workflows. Sociocultural barriers include clinician skepticism toward “black box” algorithms, differing levels of digital literacy among clinical staff, data privacy concerns, and the need for culturally and regionally tailored solutions. These challenges are not unique to HF; as highlighted by Cersosimo et al. [51], similar implementation barriers are observed in arrhythmia detection and automated echocardiography analysis. Overcoming these obstacles requires not only robust technical validation, but also strong clinician engagement, interpretable AI outputs, and effective organizational change management. In the context of coronary artery disease phenotyping, Ajiboye et al. demonstrated that collaborative interpretation and the demonstration of clinical value improve clinician trust. Collectively, these multidisciplinary experiences emphasize that efforts to address EHR integration, resource constraints, clinician education, explainability, and local adaptation are essential for scalable and sustainable ML integration in HF and across the broader spectrum of cardiovascular care.

### 8.6. Methodological Recommendations for Future Research

For future research, standardizing evaluation metrics, such as reporting AUC-ROC together with precision, recall, F1-score, and calibration plots, would enable a more complete assessment of model performance, especially with imbalanced datasets common in HF readmission studies. It is also recommended that future studies incorporate clinical text variables from discharge summaries or physician notes as well as socioeconomic and psychosocial factors to capture important predictors often missed by structured data alone. Robust external validation using data from multiple institutions or regions is essential to confirm that the models generalize beyond their developmental settings. The adoption of established reporting frameworks such as TRIPOD-AI can further improve consistency, transparency, and reproducibility. Implementing these methodological enhancements will help guide the development of more practical, reliable, and clinically meaningful ML models for predicting readmissions for HF.

### 8.7. Limitations and Future Directions

This review highlights several recurring limitations that constrain the generalizability and comparability of current ML models for HF readmission prediction (see Table 4). Most included studies relied predominantly on U.S.-based or single-center cohorts, which restricts the applicability of the findings to other healthcare systems and diverse patient populations. Dependence on administrative billing codes and structured EHR data with the infrequent inclusion of unstructured clinical notes or social determinants may result in important predictors being missed. External and multicenter validations have rarely been performed, raising concerns regarding overfitting and the robustness of model transportability. Additionally, substantial methodological heterogeneity exists, with variations in algorithm choice, predictor sets, prediction time windows, and outcome definitions, all of which complicate direct comparisons across studies. As detailed in Table 5, this review included studies published up to March 2024 and therefore did not account for more recent methodological advances, such as transformer architecture and federated learning. Addressing these limitations will require future research to incorporate multicenter and regionally diverse cohorts, expand predictor variables to include clinical texts and socioeconomic features, standardize outcome measures and reporting frameworks, and rigorously evaluate new methodological approaches. These steps are critical for the development of more robust, reliable, and equitable machine learning models for predicting HF readmissions.

## 9. Conclusions

This systematic review demonstrates ML’s potential to predict hospital readmissions among patients with HF. SLA showed a promising performance, with AUC values ranging from 0.70–0.99. However, several key areas require attention for future research and implementation. These include the need for demographic audits to address potential biases, temporal validation to account for evolving treatments, the implementation of science to bridge the gap between research and practice, standardized evaluation methods, and diverse geographic representations to enhance generalizability. Addressing these priorities will facilitate the development of more robust and equitable ML models and ultimately improve patient outcomes and reduce healthcare costs.

## Figures and Tables

**Table 1:** Summary of ML models for HF readmission prediction (2015–2022).

Study/Country	Number of Patients	% of Gender	Average Age (yrs)	Algorithm	AUC	Accuracy	Precision	Readmission Days
Allam et al. [23], USA	272,778	49 females	73	SLA	0.64	NR	NR	30 days
Angraal et al. [24], USA	1767	50 females	72	SLA	0.76	NR	NR	3-year
Frizzell et al. [7], USA	238,581	54.5 females	80	SLA	0.62	NR	NR	30 days
Golas et al. [25], USA	28,031	53 males	65	SLA	0.70	NR	NR	30 days
Jiang et al. [26], USA	534	64 females	75	ULA	0.73	NR	NR	30 days
Mahajan et al. [27], USA	1778	97.6 males	72	ULA	0.72	NR	NR	30 days
Mahajana et al. [28], USA	36,245	NA	NA	ELT	0.70	NR	NR	30 days
Pishgar et al. [29], USA	38,597	46.3 females	70	SLA	0.93	0.84	0.89	30 days
Desai et al. [30], USA	9502	45 female	78	SLA	0.76	NR	NR	1-year
Shameer et al. [31], USA	1068	NA	NA	SLA	0.78	NR	NR	30 days
Tukpah et al. [32], USA	965	NA	NA	SLA	0.69	0.78	0.58	30 days
Turgeman & May [33], USA	965	NA	79	SLA	NR	0.85	NR	30 days
Yu et al. [34], USA	20,588	NA	65	SLA	0.65	NR	NR	30 days
Sarijaloo et al. [35], USA	2441	NA	65	SLA	0.75	0.75	NR	90 days
Mortazavi et al. [10], USA	1653	NA	NA	SLA	0.67	NR	NR	180 days
Lorenzoni et al. [36], Italy	380	60 females	73	SLA	0.81	0.81	NR	1-year
Friz et al. [37], Italy	3079	55.3 females	81	SLA	0.74	0.60	0.70	30 days
Chen et al. [38], China	736	NA	72	SLA	0.67	0.67	0.71	1-year
Lv et al. [39], China	13,602	52 females	72	SLA	0.81	0.77	0.76	1-year
Bat-Erdene et al. [40], Korea	11,011	NA	NA	SLA	0.99	0.99	0.98	1-year
Awan et al. [41], Australia	10,757	49 males	81	SLA	0.62	48.42	0.70	30 days
Sharma et al. [42], Canada	9845	56 males	71	SLA	0.65	NR	NR	30 days

SLA: supervised learning algorithm; ULA: unsupervised learning algorithm; ELT: ensemble learning techniques; NR: not reported; NA: not available.

**Table 2:** Results of adapted CHARMS checklist for assessing risk of bias in included studies.

Study Name	Participant Selection	Predictor Assessment	Outcome Assessment	Model Development	Analysis
Allam et al. [23]	L	L	L	L	L
Awan et al. [41]	M	L	L	L	L
Frizzell et al. [7]	L	L	L	L	L
Golas et al. [25]	M	L	L	L	L
Jiang et al. [26]	L	L	L	L	L
Mahajan et al. [27]	L	L	L	L	L
Mahajana et al. [28]	H	L	L	L	L
Pishgar et al. [29]	L	L	L	L	L
Polo Friz et al. [37]	M	L	L	L	L
Shameer et al. [31]	L	L	L	L	L
Sharma et al. [42]	L	L	L	L	L
Turgeman & May [33]	L	L	L	L	L
Yu et al. [34]	L	L	L	L	L
Sarijaloo et al. [35]	L	L	L	L	L
Mortazavi et al. [10]	L	L	L	L	L
Bat-Erdene et al. [40]	L	L	L	L	L
Chen et al. [38]	L	L	L	L	L

Legend: L = Low; M = Medium; H = High.

**Table 3:** Comparison of key machine learning studies.

Study	Prediction Window	Sample Size	Key Findings	Strengths	Limitations	Population Diversity (% Non-White)
Allam et al. [23]	30 days	Large	Deep Learning (Neural Network), Traditional ML (Logistic Regression)	Neural networks showed slightly better performance than logistic regression for 30-day readmission risk.	Large dataset; comparison of deep learning and traditional approaches.	Homogeneous cohort; dependent on billing codes.
Frizzell et al. [7]	30 days	Large multicenter	Traditional ML (Logistic Regression, Random Forest, Gradient Boosting)	Ensemble methods did not substantially outperform logistic regression, with AUC ~0.62–0.72.	Multicenter design; rigorous validation.	U.S.-centric data; moderate discrimination.
Angraal et al. [24]	3 years	Medium	Ensemble (Random Forest)	Random forest achieved reasonable predictive power (AUC 0.76) for long-term (3-year) readmission risk.	Emphasis on long-term outcomes; advanced ML pipeline.	Relatively small/less diverse sample; limited generalizability.
Jiang et al. [26]	Dynamic (varied)	Not Reported (NR)	Unsupervised ML (Clustering: k-means)	Identified risk trajectories and clusters (e.g., “rapid decompensators”); segmentation associated with markedly different readmission risks.	Novel dynamic prediction; insight into patient heterogeneity.	Unsupervised results are harder to translate into protocols; lack of clinical actionability.
Golas et al. [25]	30 days	11,510 patients, 27,334 admissions	Traditional ML (Random Forest, Logistic Regression, SVM, Gradient Boosting)	Random forest and logistic regression had similar AUCs (0.76), supporting use of EHR data for prediction.	Large EHR dataset; real-time application design.	Limited external validation; single institution setting.
Shameer et al. [31]	30 days	Large	Traditional ML (Elastic Net Logistic Regression)	AUC 0.72; demonstrated EHR-wide ML is feasible and valuable for readmission prediction.	Comprehensive variable set; relevant to clinical workflows.	Single-center design; focus on billing code predictors.
Bat-Erdene et al. [40]	6, 12, 24 months	Moderate	Deep Learning	Deep learning outperformed traditional approaches for 6–24-month readmission prediction.	Extended follow-up window; leveraged advanced neural networks.	Lacked clinical interpretability; smaller dataset.
Chen et al. [38]	1 year	Not Reported (NR)	Deep Learning (Attention-based Neural Network)	Attention mechanisms improved interpretability and prediction with AUC 0.82.	Introduced model interpretability; highlighted features via attention weights.	Lacked comparison to other ML approaches; cohort size NR.
Lv et al. [39]	Dynamic	Not Reported (NR)	Unsupervised ML (Clustering for trajectory patterns)	High timing prediction (89% accuracy) through symptom trajectory clustering.	Focus on dynamic, interpretable trajectories; novel approach.	Hard to translate unsupervised findings into actionable clinical tools; sample size NR.
Sarijaloo et al. [35]	90 days	Moderate	Ensemble (Random Forest, Gradient Boosting)	ML models improved prediction of 90-day readmission and death versus clinical risk models.	Included robust clinical and administrative data.	Model complexity limits bedside application.

**Table 4:** Studies addressing specific limitations in HF readmission prediction.

Study	Key Strength	Critical Limitation	Clinical Limitations
Huang et al. [43]	Comprehensive scoping review of 42 studies	U.S.-centered sample (82% of included studies; no quality assessment of primary studies	Limited generalizability to non-Western healthcare systems
Angraal et al. [24]	Long-term (3-year prediction capability)	Homogeneous cohort (72% White participants); no SGLT2 inhibitor data	Underestimates risk in Asian/younger populations
Shameer et al. [31]	Health Electronic records (HER)-wide feature engineering	Single-center design; reliance on billing codes over clinical narratives	May miss social determinants affecting readmission
Allam et.al. [23]	Comparison of neural networks vs. logistic regression	Limited to 30-day readmission prediction	Provide insight on algorithm selection for short-term risk assessment
Frizzell et al. [7]	Multicenter study design	Focus on traditional statistical approaches	Establishes baseline for comparing ML to conventional methods
Jiang et al. [26]	Novel unsupervised approach for dynamic risk trajectories	Complex implementation in clinical settings	Offers new perspective on evolving readmission risk over time

**Table 5:** Comparative performance of ML models in HF readmission prediction.

Study	ML Algorithm(s) Used	Prediction Window	AUC	Key Features Used
Allam et al. [23]	Neural Network, Logistic Regression	30 days	0.64	Billing codes, labs
Frizzell et al. [7]	Random Forest, Gradient Boosting, Logistic Regression	30 days	0.62–0.72	EHR, demographics
Golas et al. [25]	Random Forest, Logistic Regression, SVM, Gradient Boosting	30 days	0.76	EHR, demographic, clinical, admission data
Chen et al. [38]	Deep Learning (Attention-based Neural Network)	1 year	0.82	EHR, text
Jiang et al. [26]	Unsupervised k-Means Clustering	Dynamic	0.73	Trajectory patterns
Shameer et al. [31]	Elastic Net Logistic Regression	30 days	0.72	EHR-wide features, billing codes
Bat-Erdene et al. [40]	Deep Learning	6, 12, 24 months	0.80–0.85	Epidemiologic, labs, admission/discharge data
Sarijaloo et al. [35]	Random Forest, Gradient Boosting	90 days	0.76	Clinical, administrative, labs
Lv et al. [39]	Unsupervised Clustering for Trajectory Patterns	Dynamic	Not Reported	Symptom trajectories
Angraal et al. [24]	Random Forest (Ensemble)	3 years	0.76	Demographic, clinical variables
Polo Friz et al. [37]	Supervised ML (Random Forest, SVM, Logistic Regression)	30 days	~0.69	LACE index, administrative, clinical

## Data Availability

This review was registered in the International Prospective Register of Systematic Reviews (ID #CRD42021247198) and contains a clear and detailed summary of the review protocol. This data is available upon request.

## Supplementary Materials

The following supporting information can be downloaded at: https://www.mdpi.com/article/10.3390/biomedicines13092111/s1.
